# Colocynth—A Lecture

**Published:** 1898-01

**Authors:** C. L. Olds


### COLOCYNTH—A LECTURE.

By C. L. Olds, M. D., H. M.

If you were to ask a dozen physicians what use they make of Colocynth, they would probably say that they use it in colic. There is another sphere of action just as important, and that is its effect on nerve-sheaths, in neuralgias. The pain is rending, burning, tearing, squeezing, cutting—the most intense kind of pain. You will find the patient excitable, very sensitive to pain. The pains are tremendous, most violent, and these pains are found throughout the body.

A prominent symptom is that the pains are brought on by anger. The patient is easily angered and extremely irritable—tears, jumps, curses, swears. We find diarrhœa from anger, vomiting from anger, suppression of the menses, or of the lochia from anger. Colic comes on from anger. After the first anger has subsided the symptoms are of such a character that they make him angry. Thus there are complaints from anger and complaints causing anger.

In the head we find the characteristic pains—rending, tearing, gnawing, cutting, pressive, especially in the left temple. The headaches may be congestive, with hot head, red face, glistening eyes. They are worse from the least motion, worse on stooping, even worse on moving the eyes; better from pressure, and usually better from heat, better in a warm room. In a general way Colocynth is relieved by heat, but there are some exceptions; the pains are sometimes better from cold. The headaches are often brought on by anger, and accompanied by difficult respiration. The patient is sensitive to a draft of air. It seems as if the skull would be torn off, so violent are the pains—all neuralgic in character; the terrible headaches may cause the patient to bend double. Sometimes with the headache there is sweat of the head smelling of urine.

In the eyes we find the same pains; iritis, with these tearing, rending, terrible pains that drive him frantic; cutting, burning, stabbing pains with acrid discharge. Aggravation from the least motion, amelioration from pressure; this is the general.

Even in the ear we find this relief from pressure. There are cutting, aching, stabbing pains, and the patient will put his fingers in the ear. Usually in earache the ear is very sensitive, but not here.

The coryza is better in the warm room, worse in the open air.

The face is pale, sallow, hippocratic, deathly—the cheeks cold. There is a left-sided neuralgia of the face, aggravated by the least touch. The pains extort cries, and are accompanied by restlessness.

There is a toothache that comes on from taking cold, from anger, driving to frenzy; it is relieved by pressure, and usually relieved by heat. *Mag-ph.* is always better from pressure, and better from heat. Colocynth is sometimes worse from heat—Mag-ph. never. The *Bryonia* toothache is better from pressure and better from *cold* things in the mouth.

In Colocynth the pains cause great restlessness; he cannot keep quiet; is worse from moving the least bit, and yet must move.

There is a very bitter taste in the mouth—everything tastes bitter. The tongue feels scalded. The appetite is at times most ravenous, or there may be little appetite. The thirst is sometimes intense. Everything they take tastes bitter, even water.

There is an empty, sinking sensation at the stomach—they must eat—and yet as surely as they eat colicy pains come on, radiating from the umbilicus as a centre, like sharp knives, or as if the intestines were ground between sharp stones. The face is pale and death-like. There is nausea and vomiting—vomiting of intensely bitter substance, bilious matter. The only relief that a Colocynth patient can get in these terrible pains is from pressure and bending over; he will bend over a chair or the bed-post, or if too weak for that will put his fists into his abdomen and bend over them. Compare *Rhus*, but Rhus is better from motion; Colocynth worse from motion. Rhus may be called a Colocynth on legs. *Dioscorea* wants the open air, and instead of bending double wants to bend back as far as he can; that relieves the pains.

The pains of Colocynth are better by passing stool or flatus. There is much noise in the abdomen, "like the croaking of frogs." The abdomen is distended with flatus. The abdominal symptoms are usually better on getting warm in bed.

We might suppose that this remedy would be useful in peritonitis or appendicitis. It seems as if the patient could not live, the pain is so intense, like the cutting of knives, or as if the intestines were ground between stones. With these symptoms, if there are cold extremities, cold sweat on the face, aggravation from motion, deadly pallor, and sinking, we find Colocynth the remedy.

The abdominal symptoms are ameliorated by coffee. Coffee will antidote Colocynth, and the patient should avoid its use.

The stools are usually copious; at first feculent, then thin, pappy, greenish, brownish, offensive, and frothy.

One of the peculiar things of this remedy is the jelly-like formations. There are stools like a jelly-fish. The urine is at first brownish, or may be white, and afterward a jelly-like mass forms in the bottom. Another sediment is reddish, sandy, and difficult to remove. The urine may be gluey or milky, and coagulating on standing.

It is useful in dysentery with the characteristic pains, bloody discharges, jelly-like stool; pieces of membrane voided. There are most intense pains before and during stools, but stool relieves. The stool sometimes looks like scrapings of intestines.

We may find in this remedy colic with a hard stool; also involuntary discharge of hard fæces.

This remedy attacks also the female sexual organs. There is inflammation of the ovaries, with pains which are worse from motion, better from eating, better from warm drinks, better from bending double, better from hard pressure. Yet after a time the abdomen becomes so tender that pressure rather aggravates than relieves. We also find inflammation of the uterus with these symptoms. There are cystic tumors with which the patient is obliged to walk bent over.

We find suppression of the menses as the result of anger. If this takes place there will almost always be a colic. So when the lochia are suppressed from anger. Even child-bed fever is cured by this remedy when it seems to have been caused by the mother's getting angry. There are colicy pains before and during the menses, and a thick, yellow, offensive leucorrhœa between.

The neuralgic pains are not confined to the upper part of the body. They go all through the body. There is sciatica, especially of the right side; pain, cutting, as from knives; worse from the least motion of the affected limb. You may find the patient in bed with the affected limb flexed on the abdomen, with terrible pains going down the limb from the back, unable to straighten out the limb. One patient who was cured by a single dose had the limb doubled up and pressed on the abdomen; this relieved the pains.

Colocynth cures hip-joint disease with these terrible pains.

In the colic of different animals—*e. g.*, the horse—nine times out of ten Colocynth is the remedy. The horse tries to draw its legs up if standing, or, if lying, will draw up into a ball, and will roll about the stable in the most pitiful manner. Colocynth will nearly always cure. But if after eating much clover the abdomen is much distended, with great pains and passing of wind, then *Colchicum* is the remedy. So in the human family, in colic after eating green things, as cabbage or lettuce.

Colocynth is sometimes indicated in lead colic.

